# A universal predictive and mechanistic urinary peptide signature in acute kidney injury

**DOI:** 10.1186/s13054-022-04193-9

**Published:** 2022-11-07

**Authors:** Alexis Piedrafita, Justyna Siwy, Julie Klein, Amal Akkari, Ana Amaya-garrido, Alexandre Mebazaa, Anna Belen Sanz, Benjamin Breuil, Laura Montero Herrero, Bertrand Marcheix, François Depret, Lucie Fernandez, Elsa Tardif, Vincent Minville, Melinda Alves, Jochen Metzger, Kidney Attack Study Group, Etienne Grunenwald, Guylène Feuillet, Marie Buléon, Manon Brunet, Nicolas Mayeur, Audrey Casemayou, François Labaste, Julia Grossac, Harald Mischak, Alberto Ortiz, Stéphane Gazut, Joost P. Schanstra, Stanislas Faguer

**Affiliations:** 1 Department of Nephrology and Organ Transplantation, University Hospital of Toulouse, and French Intensive Care Renal Network, 31000 Toulouse, France; 2 National Institute of Health and Medical Research (INSERM), UMR 1297, Institute of Cardiovascular and Metabolic Disease, 31000 Toulouse, France; 3 University Paul Sabatier, Toulouse-III, 31000 Toulouse, France; 4 Mosaiques Diagnostics GmbH, Hannover, Germany; 5 Université Paris-Saclay, CEA, List, 91120 Palaiseau, France; 6 Department of Anesthesiology, Critical Care and Burn Unit, Hôpitaux Universitaires Saint Louis-Lariboisière, Assistance Publique-Hôpitaux de Paris, Université Paris Diderot-Paris 7, Sorbonne Paris Cité, UMR-S 942, INSERM, France, INI-CRCT, ParisNancy, France; 7 School of Medicine, IIS-Fundación Jiménez Díaz, Autonomous University of Madrid, FRIAT and REDINREN, Madrid, Spain; 8 Department of Cardiac and Vascular Surgery, University Hospital of Toulouse, 31000 Toulouse, France; 9 Department of Anesthesiology and Critical Care Medicine, University Hospital of Toulouse, 31000 Toulouse, France

**Keywords:** Acute kidney injury, Cardiac surgery, Intensive care unit, Urinary peptidomics, Prediction

## Abstract

**Background:**

The delayed diagnosis of acute kidney injury (AKI) episodes and the lack of specificity of current single AKI biomarkers hamper its management. Urinary peptidome analysis may help to identify early molecular changes in AKI and grasp its complexity to identify potential targetable molecular pathways.

**Methods:**

In derivation and validation cohorts totalizing 1170 major cardiac bypass surgery patients and in an external cohort of 1569 intensive care unit (ICU) patients, a peptide-based score predictive of AKI (7-day KDIGO classification) was developed, validated, and compared to the reference biomarker urinary NGAL and NephroCheck and clinical scores.

**Results:**

A set of 204 urinary peptides derived from 48 proteins related to hemolysis, inflammation, immune cells trafficking, innate immunity, and cell growth and survival was identified and validated for the early discrimination (< 4 h) of patients according to their risk to develop AKI (OR 6.13 [3.96–9.59], *p* < 0.001) outperforming reference biomarkers (urinary NGAL and [IGFBP7].[TIMP2] product) and clinical scores. In an external cohort of 1569 ICU patients, performances of the signature were similar (OR 5.92 [4.73–7.45], *p* < 0.001), and it was also associated with the in-hospital mortality (OR 2.62 [2.05–3.38], *p* < 0.001).

**Conclusions:**

An overarching AKI physiopathology-driven urinary peptide signature shows significant promise for identifying, at an early stage, patients who will progress to AKI and thus to develop tailored treatments for this frequent and life-threatening condition. Performance of the urine peptide signature is as high as or higher than that of single biomarkers but adds mechanistic information that may help to discriminate sub-phenotypes of AKI offering new therapeutic avenues.

**Supplementary Information:**

The online version contains supplementary material available at 10.1186/s13054-022-04193-9.

## Background

Acute kidney injury (AKI) is a life-threatening disease with an incidence of 13.5 million patients and an estimated 1.7 million deaths per year worldwide [1]. In survivors, the risk of chronic kidney disease (CKD) increases ninefold [2]. Worldwide, more than 850 million people suffer from AKI and CKD or require renal replacement therapy (RRT) [1]. There is a clear need for early detection (< 12 h after the injury) to reduce the severity of the AKI. However, despite recent and intensive efforts, AKI is still detected at a late stage [1 to 3 days after the injury]. In addition, although treatments exist to reduce the impact of full-blown AKI, they are not specific and do not focus on the molecular mechanisms of AKI. The International Society of Nephrology has formally recognized this alarming situation of AKI as a major challenge and has launched the “0by25” objective, to eliminate preventable deaths from AKI by 2025 in low- and high-income countries [1], echoed by the European Renal Association [3].

The late detection of AKI is largely related to the assessment of kidney dysfunction by serum creatinine, which is inherently downstream of advanced AKI, rather than based on earlier signs of kidney damage [4–8]. Furthermore, even the recently discovered individual molecular markers of kidney injury (*e.g.*, FGF-23, NGAL, IL-18, KIM-1, [TIMP-2].[IGFBP7]) detect AKI optimally at 12–24 h post-injury, when often irreversible damage is already present [4–7, 9]. These individual biomarkers are mainly dependent on the injury to a specific tubular segment, appear non-specific, and do not provide information on the timing and mechanisms of kidney injury [4–8]. To grasp the molecular complexity of AKI, high-throughput strategies using multi-dimensional molecular markers should therefore be proposed, as already employed in other acute conditions such as acute respiratory distress syndrome, acute heart failure, or sepsis [10–16].

Urinary peptidomics has emerged as a powerful method to noninvasively assess characteristics of the kidney parenchyma [17, 18] and to stratify patients according to their risk of progressing to kidney fibrosis [19–21], but also to assess specific risks of systemic diseases, including acute life-threatening conditions such as COVID-19 [22]. In the context of AKI, early preliminary studies using a small number of patients (*n* = 80–120) have shown the feasibility of using urinary peptidome analysis to predict the development of AKI, outperforming NGAL (neutrophil gelatinase-associated lipocalin) and KIM-1 (kidney injury molecule-1) [23, 24]. However, the samples were collected at a late stage after kidney injury.

In the current study, we determined whether urinary peptide signatures can identify, at an early stage, patients developing AKI in different at-risk clinical settings including cardiac bypass (CBP) surgery and after admission to the intensive care unit (ICU) and grasp the molecular complexity of AKI to identify potential targetable pathways.

## Methods

### Study design

A multi-step strategy was developed to identify a potential urinary peptide signature of AKI after CBP surgery. First, clinical characteristics were assessed in a derivation cohort of patients referred for cardiac surgery with cardiac bypass and used to identify clinically derived predictive factors of AKI. Urinary peptidomes were then characterized using mass spectrometry, and differentially abundant peptides between AKI and non-AKI patients with a significant Benjamini–Hochberg adjusted Wilcoxon signed-rank testing were identified. The link of one of the peptides to the AKI pathophysiology was confirmed by studying the full-length protein (calprotectin) in urine, in an animal AKI model and *in vitro*. A set of sequenced peptides was used to build a support vector machine-based predictor. Results were extracted as a calibrated score based on the derivation and then tested in two validation cohorts of patients referred for CBP surgery or admitted to the general ICU.

### Patients

CBP surgery patients were prospectively recruited at the University Hospital of Toulouse, France, during two distinct time periods (March 2016–January 2017 for the derivation cohort (*n* = 509) and January 2019–March 2020 for the validation cohort *n* = 661). All patients with CBP surgery were eligible. Patients under 18, that underwent unscheduled CBP surgery or who required chronic dialysis before surgery were excluded.

A third cohort of patients admitted to the general ICU were also studied to obtain external validation of the peptide signature. Detailed clinical characteristics of this European multicenter cohort that included 1569 patients admitted to the ICU for sepsis, heart failure, cardiac arrest, or urgent surgery were already reported in previous studies (FROG-ICU) [25–27]. Urine samples were collected during the first 24 h following admission. AKI KDIGO classification [7 days period] was used to define AKI severity.

### Characteristics, definitions, and endpoints

Pre-, per-, and postoperative clinical data were gathered retrospectively for all patients based on hospital records. Baseline estimated glomerular filtration rate (eGFR) was estimated using the CKD-EPI formula based on standardized creatinine measurement (IDMS) before cardiac surgery [28]. EuroSCORE-II was calculated as recommended [29].

Surgery was divided into coronary artery bypass (CAB), valvular surgery (valvuloplasty or replacement), combined CAB and valvular surgery, surgery with replacement of the ascending aorta with or without CAB (aortic surgery); surgery that directly affects the cardiac myocardial wall, such as interatrial communication, interventricular communication, ventricular aneurism, or cardiac transplantation (myocardium).

The main endpoint, AKI, was defined according to the AKI kidney disease/improving global outcome (KDIGO) 2012 criteria [30] evaluated during the first 7 days after surgery. Briefly, AKI was defined as a significant increase in serum creatinine (> 1.5 times baseline or > 26.5 μmol/L increase) or a reduced urine output (< 0.5 mL/kg/h for at least 6 h) or RRT requirement. In the external ICU cohort (FROG-ICU cohort), AKI definition only relied on the serum creatinine criteria [25, 26].

CSA-AKI, Ng, Cleveland, AKICS, and SRI scores were calculated as described [31–35]. Some parameters were approximated as follows: History of congestive heart failure was approximated as left ventricular ejection fraction (LVEF) < 60%; preoperative capillary glucose was assimilated with diabetic status regardless of the treatment received; central venous pressure was considered the maximum pressure during the first 24 h after surgery; and low cardiac output was defined as the need for vasopressive or inotropic drugs.

### Urinary peptidome analysis

In the CBP surgery cohorts, urine samples were collected 2.5 to 4 h after surgery and immediately frozen (− 20 ℃) before subsampling and re-frozen for long-term conservation (− 80 ℃). In the external ICU validation cohort, urine was collected in the first 24 h of admission to the ICU, immediately frozen (− 20 ℃) before subsampling and re-frozen for long-term conservation (− 80 °C).

Peptide extraction and CEMS processing were performed as previously described (the extended methodology is given in Additional file 1: S1) [36].

### Development of peptide-based score

For peptidome analysis, among the 5862 peptides, only peptides with less than 70% of missing data in at least one group were considered for analysis, resulting in a set of 1255 peptides. For those peptides, missing values were replaced by 0 before further analysis. Univariate testing between AKI and non-AKI patients was performed using the Wilcoxon signed-rank test, followed by Benjamini–Hochberg false discovery rate adjustment. Correlations were performed according to the Pearson method.

For score derivation, all patients from the CBP surgery discovery cohort with available peptidome data were considered (*n* = 446). A set of 328 differentially abundant peptides with a significant Benjamini–Hochberg adjusted Wilcoxon signed-rank testing was identified in this cohort. The amino acid sequence could be obtained for 204 peptides of the 328 peptides. These 204 peptides were used to build a support vector machine-based predictor (MosaCluster software [37]). Results were extracted as a calibrated score based on derivation. Score performances in the CBP surgery derivation cohort were estimated using the leave-one-out procedure. Score validation was then obtained in the CBP surgery validation cohort in patients with available peptidome data; *n* = 480. External validation in the ICU context was obtained on all patients from the FROG-ICU cohort with available AKI status and peptidome data (*n* = 1569).

Additional statistical analyses, including scores comparisons and the use of genetic algorithms, are described in Additional file 1: text S1.

### Urine NGAL, IGFBP7, TIMP2, calprotectin, and creatinine measurement

Urine samples were centrifuged for 10 min at 2500 rpms. NGAL was measured using ELISA (Human Lipocalin-2/NGAL DuoSet ELISA, R&D, DY1757) in diluted supernatant (1/10 or 1/100) according to the manufacturer’s protocol. Calprotectin was measured using ELISA (Human S100A8/S100A9 Heterodimer DuoSet ELISA, DY8226-05) according to the manufacturer protocol. Creatinine was measured using the QuantiChrom Creatinine Assay Kit (BioAssay Systems, DICT 500) according to the manufacturer protocol. Creatinine-normalized NGAL and creatinine-normalized calprotectin concentrations (microg/g) were used for performance evaluations. TIMP-2/IGFBP7 was measured in urine supernatants using the VITROS NephroCheck immunoassay on a VITROS 5600 Integrated System (Ortho Clinical Diagnostics) according to the manufacturer’s instructions. The Vitros NephroCheck Test result is a single numerical, which is a product of the measured concentrations of the two analytes in the sample divided by 1000.

Certified laboratory technicians blinded to clinical data performed the analyses.

### Mouse model of ischemic AKI

Ischemic AKI was induced in C57Bl6 male mice using warm renal ischemia/reperfusion (bilateral clamping of renal arteries for 20 min) (see Additional file 1: text S1). Kidney samples were analyzed and blood urea nitrogen was measured at 6, 24, and 48 h.

Animal experiments were approved by the local and national ethical committees (CREFRE Inserm/UPS, agreement C31 55,507; Protocol APAFIS#122–2015-23).

### Cell culture

MCT cells were grown under standard conditions (21% O_2_, 5% CO_2_, 37 °C) and submitted to hypoxia for 48 h or TNFα or IL-1*β* exposure for 8 h. mRNA of KIM1 and calprotectin was quantified in each condition using quantitative polymerase chain reaction (see Additional file 1: Text S1).

### Statistics

Statistical analyses were performed with the R (v4.0.3) software using RStudio interface with additional packages caret [38], pROC [39], ROCR [40], mgcv [41], and GA [42]. Detailed statistical procedures can be found in the Additional file 1: text S1.

*Study approval*: All the patients were orally informed of the inclusion during the anesthetic consultation performed during the weeks before surgery and the non-opposition of the patients to being included in the clinical and biological collection of the University Hospital of Toulouse was obtained before inclusion (agreement number French national ethical committee DC-2008–463). The study was performed according to the Declaration of Helsinki, as revised in 2004. Patients in the external ICU validation cohort were included in the Frog-ICU cohort [25–27].

## Results

### Cardiac surgery cohorts and AKI incidence

**Fig. 1:**
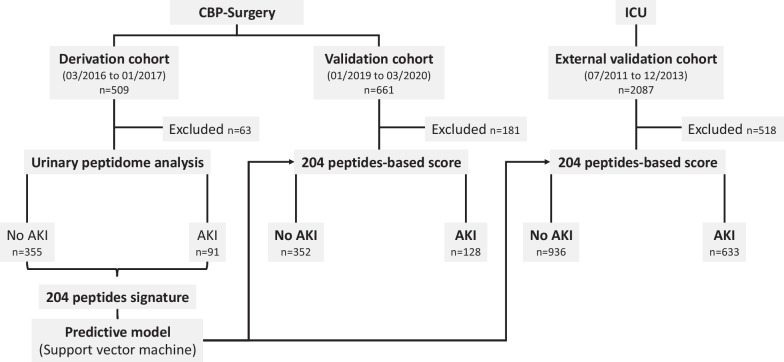
Patient flowchart for the identification and validation of a predictive AKI urinary peptide signature. Three independent cohorts were used: a derivation CBP surgery cohort (*n* = 509), a validation CBP surgery cohort (*n* = 661)—both recruited in the University Hospital of Toulouse (France), but during different time periods—and an external ICU cohort (external ICU validation multicenter cohort [25], *n* = 2087). Sixty-three patients from the derivation and 181 from the validation CBP surgery cohorts were excluded because of missing urine samples or failure of the urinary peptidome analysis pipeline. Five hundred eighteen patients from the external ICU validation cohort were excluded because of missing urine samples, failure of the urinary peptidome analysis pipeline, or missing information with respect to the development or presence of AKI. *CBP surgery*, cardiac bypass surgery; *ICU*, intensive care unit

We prospectively included a total of 1170 patients (mean age 66.7 ± 12.0 years) referred for CBP surgery in a single center (Toulouse, France). Inclusion was performed during two different time periods resulting in two independent cohorts: a derivation cohort (*n* = 509) with patients included between March 2016 and January 2017 and a validation cohort (*n* = 661) with patients included between January 2019 and March 2020 (Fig. 1).

Patient characteristics are summarized in Table 1. Patients from the validation cohort had better baseline kidney function (eGFR 77.6 ± 20.6 vs. 70.3 ± 20.2 mL/min/1.73m^2^, *p* < 0.001), were more frequently affected by hypertension (*p* = 0.029) and chronic obstruction pulmonary disease (COPD) (*p* < 0.001) and had more frequently undergone previous cardiac surgery (*p* = 0.022). Due to anesthetic procedure changes between the two inclusion periods, the number of red blood cell (RBC) transfusions during surgery was lower (*p* = 0.001) and the use of vasoactive agents was more frequent (*p* = 0.003) in the validation cohort. During the postoperative period, patients included in the validation cohort received iodine contrast agents less frequently (*p* = 0.001) and had a shorter length of stay within the ICU (*p* < 0.0001). However, this did not lead to significant differences in AKI incidence between the derivation and validation cohort (~ 23%, KDIGO stage 1–3). Severe AKI (KDIGO stage 2 or 3) was identified in 42 (8.3%) and 93 (14.0%) patients, respectively. Eighteen (3.5%) and 14 (2.1%) patients required RRT in the derivation and validation cohorts, respectively.

**Table 1:** Characteristics of patients included in the derivation and validation CBP cohorts

Parameters CBP cohort	Derivation (*n* = 509)	Validation (*n* = 661)	Adjusted univariate *p* value
Preoperative features			
Male, *n* (%)	381 (74.9)	501 (75.8)	0.836
Age (years), mean ± SD	67.6 ± 11.4	66.1 ± 12.4	0.071
BMI (kg/m^2^), mean ± SD	26.9 ± 4.4	26.8 ± 4.7	0.811
Diabetes, *n* (%)	127 (25)	179 (27.1)	0.571
Hypertension, *n* (%)	269 (52.8)	399 (60.4)	0.029
PAOD, *n* (%)	49 (9.6)	59 (8.9)	0.836
Stroke, *n* (%)	33 (6.5)	57 (8.6)	0.312
COPD, *n* (%)	29 (5.7)	97 (14.7)	< 0.001
EuroSCORE-II, mean ± SD	2.4 ± 2.6	2.7 ± 4.3	0.150
LVEF (%), mean ± SD	55.9 ± 11.3	56.1 ± 11	0.844
Serum Creatinine (μmol/L), mean ± SD	98.3 ± 41.2	88.3 ± 30.1	< 0.001
eGFR (mL/min.1.73m^2^), mean ± SD	70.3 ± 20.2	77.6 ± 20.6	< 0.001
Kidney graft recipients, n (%)	7 (1.4)	6 (0.9)	0.771
Per-operative features			
Surgery			
CAB, *n* (%)	196 (38.5)	245 (37.1)	
Valvular, *n* (%)	175 (34.4)	199 (30.1)	0.220
Combined, *n* (%)	71 (13.9)	105 (15.9)	
Thoracic aorta, *n* (%)	58 (11.4)	87 (13.2)	
Myocardium, *n* (%)	9 (1.8)	25 (3.8)	
Previous cardiac surgery, *n* (%)	23 (4.5)	57 (8.6)	0.022
CBP time (min), mean ± SD	85.3 ± 36.2	88.5 ± 36.5	0.246
RBC transfusion, *n* (%)	91 (17.9)	59 (8.9)	< 0.001
Number, mean ± SD	0.4 ± 0.9	0.2 ± 0.8	0.001
Vasoactive agents, *n* (%)	456 (89.6)	627 (94.9)	0.003
Postoperative features			
RBC transfusion, *n* (%)	151 (29.7)	155 (23.4)	0.045
Number, mean ± SD	0.8 ± 1.8	0.7 ± 1.9	0.368
Vasoactive agents, *n* (%)	277 (54.4)	445 (67.3)	< 0.001
Duration (day), mean ± SD	1.2 ± 2.1	1.6 ± 2.6	0.004
Infection, *n* (%)	83 (16.3)	112 (16.9)	0.858
Iodinated contrast agents, *n* (%)	25 (4.9)	7 (1.1)	0.001
Mechanical ventilation duration (d), mean ± SD	19.5 ± 77.2	12.4 ± 42.6	0.122
ICU stay duration (day), mean ± SD	5.6 ± 6.7	4.2 ± 5.2	< 0.001
Outcomes			
AKI KDIGO			
0, *n* (%)	389 (76.4)	486 (73.5)	
1, *n* (%)	78 (15.3)	82 (12.4)	0.005
2, *n* (%)	23 (4.5)	69 (10.4)	
3, *n* (%)	19 (3.7)	24 (3.6)	
In-hospital mortality, *n* (%)	15 (2.9)	26 (3.9)	0.571

This table presents the clinical characteristics and outcome of all CBP patients irrespective of the availability of a urine sample.

*BMI* Body mass index, *PAOD* Peripheral artery obliterans disease, *COPD* Chronic obstructive pulmonary disease, *LVEF* Left ventricular ejection fraction, *eGFR* Estimated glomerular filtration rate, *CBP* Cardiac bypass, *RBC* Red blood cells, *SD* Standard deviation, *AKI* Acute kidney injury, *KDIGO* Kidney disease/improving global outcome classification

### Pre- and per-operative clinical parameters are moderate predictors of AKI after CBP surgery

In the 509 patients of the derivation cohort, the univariate analysis identified several clinical features that were associated with the development of AKI (Table 2). These included preoperative (age, hypertension, kidney transplantation, baseline eGFR) and per-operative (CBP surgery length, RBC transfusion) parameters. The majority remained significantly associated with the development of AKI after multivariate logistic regression adjustment.

**Table 2:** Predictive factors of AKI after CBP surgery (derivation cohort, *n* = 509)

Parameters (derivation CBP cohort)	Overall (*n* = 509)	Acute kidney injury			
		No (*n* = 389)	Yes (*n* = 120)	Adjusted univariate *p* value	Multivariate odds ratio (95% CI)
*Preoperative features*					
Male, *n* (%)	381 (74.9)	295 (75.8)	86 (71.7)	0.504	–
Age (years), mean ± SD	67.6 ± 11.4	66.7 ± 12	70.3 ± 9.1	0.002	1.03 [1.00–1.05]
BMI (kg/m^2^), mean ± SD	26.9 ± 4.4	26.8 ± 4.2	27.5 ± 5.1	0.237	–
Diabetes, *n* (%)	127 (25)	93 (23.9)	34 (28.3)	0.492	–
Hypertension, *n* (%)	269 (52.8)	194 (49.9)	75 (62.5)	0.033	1.55 [0.98–2.50]
PAOD, *n* (%)	49 (9.6)	32 (8.2)	17 (14.2)	0.116	1.34 [0.63–2.72]
Stroke, *n* (%)	33 (6.5)	25 (6.4)	8 (6.7)	1.000	–
COPD, *n* (%)	29 (5.7)	23 (5.9)	6 (5)	0.911	–
EuroSCORE-II, mean ± SD	2.4 ± 2.6	2.1 ± 2.2	3.4 ± 3.3	< 0.001	–
LVEF (%), mean ± SD	55.9 ± 11.3	56 ± 11.4	55.7 ± 11.2	0.902	–
Serum Creatinine (μmol/L), mean ± SD	98.3 ± 41.2	92.5 ± 25.1	117.4 ± 68.7	0.001	–
eGFR (mL/min.1.73m^2^), mean ± SD	70.3 ± 20.2	72.9 ± 18.3	61.8 ± 23.7	< 0.001	0.80 [0.71–0.91]
Kidney graft recipients, *n* (%)	7 (1.4)	1 (0.3)	6 (5)	0.002	18.75 [2.64–385.1]
*Per-operative features*					
Surgery				0.504	–
CAB, n (%)	196 (38.5)	156 (40.1)	40 (33.3)		
Valvular, n (%)	175 (34.4)	135 (34.7)	40 (33.3)		
Combined, n (%)	71 (13.9)	50 (12.9)	21 (17.5)		
Thoracic aorta, *n* (%)	58 (11.4)	41 (10.5)	17 (14.2)		
Myocardium, *n* (%)	9 (1.8)	7 (1.8)	2 (1.7)		
Previous cardiac surgery, *n* (%)	23 (4.5)	13 (3.3)	10 (8.3)	0.062	1.23 [0.39–3.65]
CBP time (min), mean ± SD	85.3 ± 36.2	79.3 ± 30.8	104.8 ± 44.8	< 0.001	1.02 [1.01–1.03]
RBC transfusion, *n* (%)	91 (17.9)	56 (14.4)	35 (29.2)	0.001	0.50 [0.003–3.33]
Number, mean ± SD	0.4 ± 0.9	0.3 ± 0.8	0.7 ± 1.2	0.003	1.34 [0.61–2.93]
Vasoactive agents, n (%)	456 (89.6)	345 (88.7)	111 (92.5)	0.403	–
*Postoperative features*					
RBC transfusion, *n* (%)	151 (29.7)	90 (23.1)	61 (50.8)	< 0.001	–
Number, mean ± SD	0.8 ± 1.8	0.5 ± 1.1	1.8 ± 2.9	< 0.001	–
Vasoactive agents, *n* (%)	277 (54.4)	194 (49.9)	83 (69.2)	0.001	–
Duration (day), mean ± SD	1.2 ± 2.1	0.9 ± 1.3	2.2 ± 3.6	0.001	–
Infection, *n* (%)	83 (16.3)	44 (11.3)	39 (32.5)	< 0.001	–
Iodinated contrast agents, n (%)	25 (4.9)	12 (3.1)	13 (10.8)	0.003	–
*Mechanical ventilation*					
Duration (*d*), mean ± SD	19.5 ± 77.2	11.1 ± 35.1	46.9 ± 143.4	0.014	–
ICU stay duration (day), mean ± SD	5.6 ± 6.7	4.6 ± 3.8	8.8 ± 11.4	0.001	–
In-hospital mortality, *n* (%)	15 (2.9)	1 (0.3)	14 (11.7)	< 0.001	–

*BMI* Body mass index, *PAOD* Peripheral artery obliterans disease, *COPD* Chronic obstructive pulmonary disease, *LVEF* Left ventricular ejection fraction, *eGFR* Estimated glomerular filtration rate, *CBP* Cardiac bypass, *RBC* Red blood cells, *SD* Standard deviation, *AKI* Acute kidney injury, *KDIGO* Kidney disease/improving global outcome classification

**Fig. 2:**
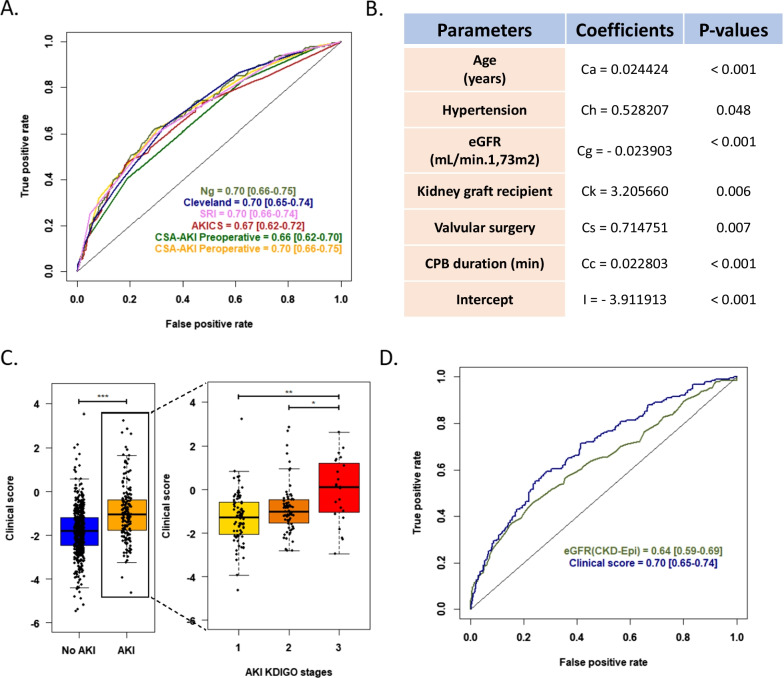
AKI prediction in CBP surgery patients based on clinical pre- and per-operative features. **A.** ROC curves and corresponding AUROC [95% confidence interval] of published clinical scores for the prediction of AKI (KDIGO 1, 2, or 3) in the CBP surgery validation cohort. **B.** Parameters and associated coefficients of a local clinical model defined in the derivation cohort. The clinical score is calculated as follows: logit(p(AKI)) = Ca x Age (Years) + Ch x Hypertension (0/1) + Cg x eGFR (mL/min.1.73m^2^) + Ck x Kidney Graft Recipient (0/1) + Cs x Valvular Surgery (0/1) + Cc x CPB Duration (min) + I. **C.** Association of the local clinical score with the development of AKI in the validation cohort (all stages, left; according to KDIGO stages, right). * *p* < 0.05; ** *p* < 0.01; *** *p* < 0.001. **D.** ROC curves and corresponding AUROC [95% confidence interval] of the local clinical score compared to baseline eGFR for the prediction of all stages of AKI in the validation cohort. The AUROCs of the local clinical score and eGFR were significantly different (Delong test; *p* value = 0.007). *ROC*, receiver operating characteristic curve; *AUROC*, area under the receiver operating characteristic curve*; CI*, confidence interval; *AKI*, acute kidney injury; *CBP*, cardiac bypass; *eGFR*, estimated glomerular filtration rate

These parameters, both used as single markers (*e.g.*, eGFR) or included in more complex, previously published scores (*i.e.*, CSA-AKI [31], Ng [32], Cleveland [33], AKICS [34] or SRI [35] scores) had poor discriminative power (area under the received operating curves (AUC) of 0.64–0.70 in the validation cohort) (Fig. 2). We therefore built a local clinical score using six pre- and per-operative variables identified by stepwise selection in the derivation cohort (age, hypertension, eGFR, kidney transplantation, valvular surgery, and CBP surgery length) (Fig. 2). However, although there was a clear difference in the local score of patients developing AKI (*p* < 0.001), this score was not significantly better than the previously established scores (*p* > 0.1 for all). It is therefore evident that complementary strategies are needed to improve the prediction of patients developing AKI following CBP surgery at an early stage.

### AKI leads to early changes in the urinary peptidome

**Fig. 3:**
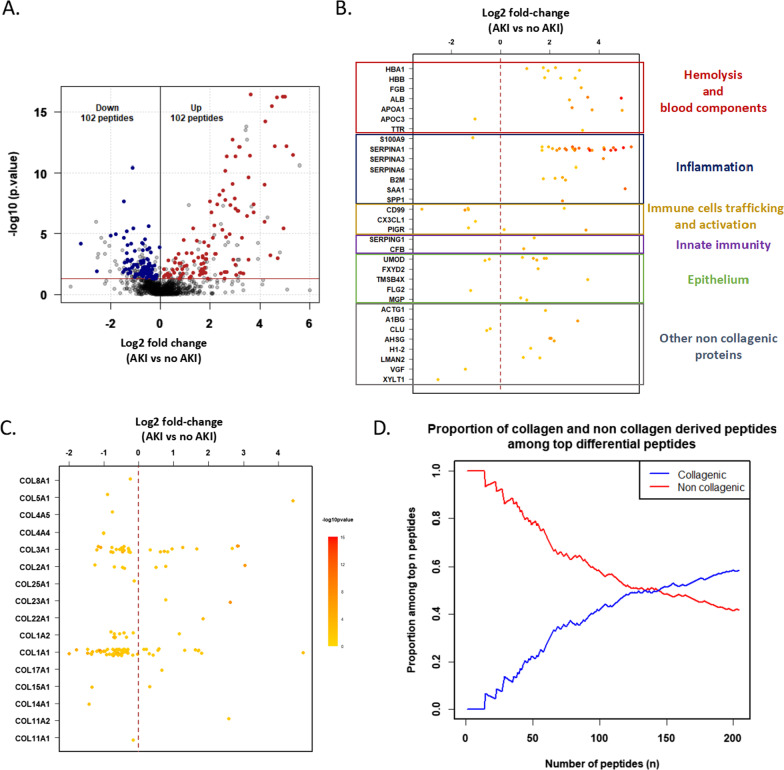
Urinary peptidome changes during CBP-surgery-induced AKI. **A**. Peptides with significantly different abundances in AKI patients. The volcano plot displays log10-transformed and adjusted univariate *p* values as a function of log2-transformed fold changes of urinary peptides amplitudes. Sequenced peptides with differential abundances (significant after Benjamini–Hochberg adjusted Wilcoxon univariate testing (*p* < 0.05)) are represented in color (more abundant: red; less abundant: blue). **B**. Peptides with significantly different abundances (in respect to distribution frequency and amplitude signal) derived from non-collagenic proteins (y position), log2 fold changes (x position), and –log10 adjusted *p* values (color scale). The brown dashed line represents log2 fold change = 0. Peptides are ranked according to their functional role during the AKI progression (inflammation, epithelium, blood component, other non-collagenic proteins). **C**. Peptides with significantly different abundances derived from collagenic proteins (y position), log2 fold changes (x position), and –log10 adjusted p values (color scale). The brown dashed line represents log2 fold change = 0. **D**. Collagenic and non-collagenic proteins-derived peptides proportions among top differential peptides according to the Benjamini–Hochberg adjusted *p* value ranking. The red line corresponds to non-collagen and the blue line to collagen-derived peptides

We analyzed for the first time at a very early stage (< 4 h after CBP surgery) the urinary peptide content in this large CBP surgery cohort to identify candidate biomarkers predictive of AKI that could help with clinical decision-making and stratifying patients for further interventional studies. Using the 509 patients in the derivation cohort, 204 displayed a significantly different abundance (Benjamini–Hochberg adjusted Wilcoxon univariate testing (*p* < 0.05)) in the urine of patients developing AKI (up-regulated *n* = 102; down-regulated *n* = 102; Fig. 3). Peptides associated with AKI were derived from 48 proteins, including 16 collagens and 32 non-collagenous proteins. Interestingly, when ranked according to their adjusted univariate p values, top differential peptides were mainly derived from non-collagenous proteins (Fig. 3). These non-collagenous proteins were related to hemolysis (*e.g.*, hemoglobin α and *β*-subunit), inflammation (*e.g.*, S100A9 (a calprotectin subunit), SERPINA1 (*α*1-antitrypsin), B2M (*β*2-microglobulin)), immune cell trafficking and activation (*i.e.*, CD99, CX3CL1, PIGR), innate immunity (*e.g.*, complement factor B), kidney epithelium (*e.g.*, UMOD (uromodulin), FXYD2) and cell growth and survival (*e.g.*, ACTG1).

To confirm the relevance of the urinary signature, we measured the expression of S100A9, alone or combined in the form of calprotectin (S100A8/S100A9) in in vitro and in vivo models of epithelial injuries, as well as in the urine of CBP surgery patients. As shown in Additional file 1: Fig. S1, following cardiac surgery, calprotectin was increased in the urine of patients who will develop AKI (Fig. S1A). In C57Bl/6 mice, 20-min bilateral renal ischemia/reperfusion injury induced AKI characterized by increased BUN. In mice, AKI was associated with the upregulation of renal *S100A9* mRNA expression as well as S100A9 protein expression within the kidney cortico-medullary junction (Fig. S1B-E). Renal expression of the kidney injury molecule *Kim1* was also increased in this model (Fig. S1D). Finally, in mouse proximal tubule cells (MCT cell line), 48 h of hypoxia and/or 8 h exposure to the pro-inflammatory cytokine tumor necrosis factor-*α* or interleukin-1*β* induced *S100a9* and *Kim1* mRNA expression (Fig. S1G-J).

### A urinary peptide-based signature predicts CBP-surgery-induced AKI and significantly outperforms clinical parameters.

**Fig. 4:**
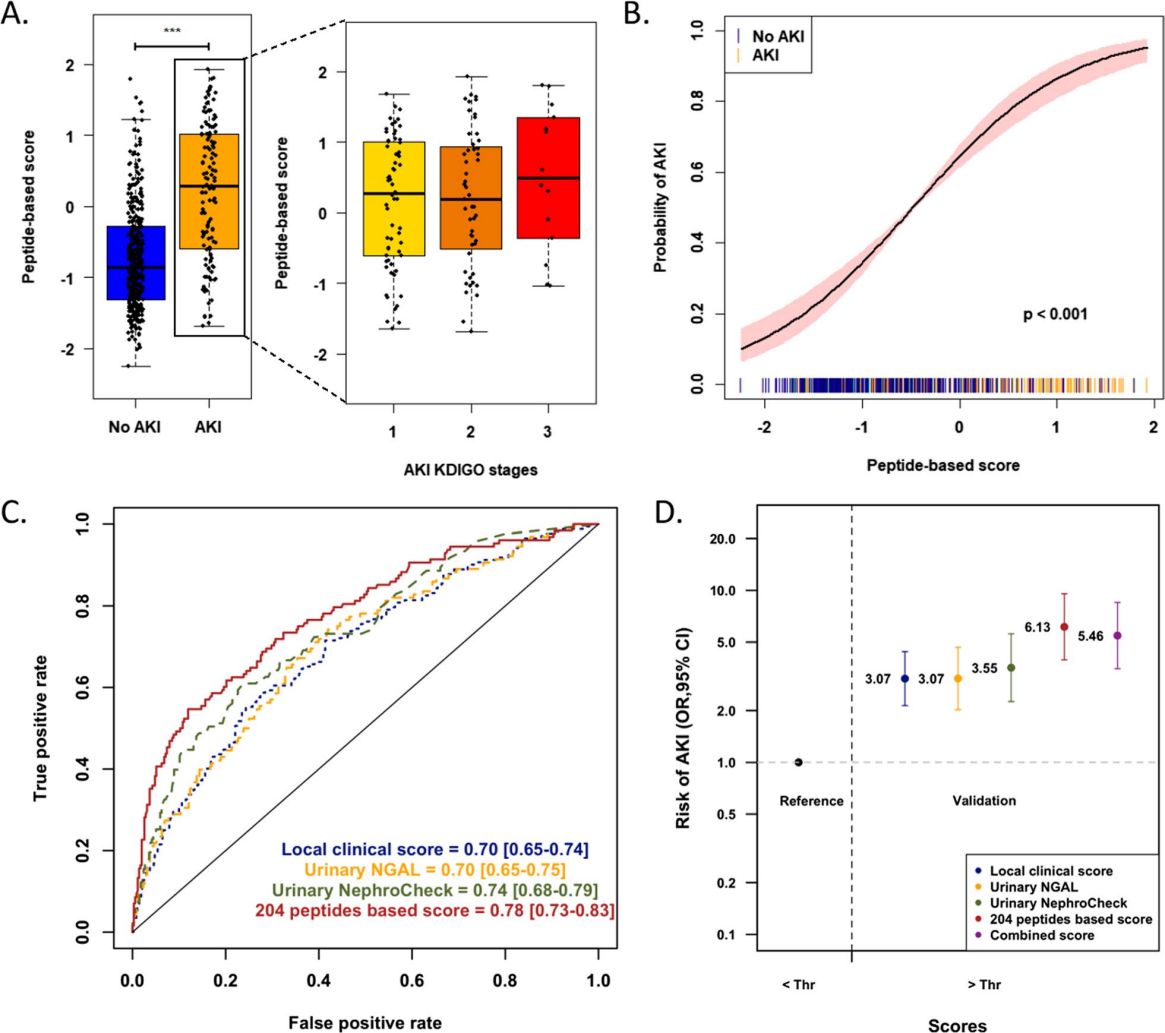
Independent validation of the predictive value of the urinary peptide signature for early AKI diagnosis after CBP surgery. **A** Association of the urinary peptide-based score with the development of AKI (all stages, left; according to KDIGO stages, right) in the CBP surgery validation cohort (*n* = 480). *** *p* < 0.001. **B** Spline plot of the association between the peptide-based score and the risk of developing AKI. A univariate logistic generalized additive model was used. The black line indicates the estimated risk of AKI with respective 95% confidence intervals. The spikes show the distribution of the peptide-based scores. **C** ROC curves with corresponding AUROC and 95% confidence intervals for the 204 peptides-based score, the local clinical score (and reference urinary AKI biomarkers NGAL and TIMP2*IGFBP7 for the prediction of AKI (all stages) in the validation cohort. **D** Odds ratios and corresponding 95% confidence intervals in the validation cohort using a multivariate logistic regression model including the local clinical score, reference urinary AKI biomarkers NGAL and TIMP2*IGFBP7, the 204 peptides-based score or a combination of the local clinical and peptide-based scores as a qualitative value according to the selected threshold (optimal Youden index in the derivation cohort). *ROC*, receiver operating characteristics curve; *AUROC*, area under the receiver operating characteristics curve*; CI*, confidence interval; *Thr*, threshold

Next, the 204 peptides were included using machine learning in a support vector machine-based mathematical model that was trained on the derivation cohort and applied in the validation cohort. In the validation cohort, the signature clearly identified patients developing AKI (*p* < 0.001), with an increase in AKI risk at a higher peptide-based score (*p* < 0.001; Fig. 4).

Pre- and per-operative characteristics (diabetes mellitus, COPD, baseline eGFR, surgery indication, redux surgery, CBP surgery length) partly correlated with the peptide-based score (Additional file 1: Table S1). However, the urinary peptide signature contained complementary information with respect to the clinical characteristics, as evidenced by (*i*) an adjusted R-squared of the multivariate model of 0.246, (*ii*) the significant correlation between AKI development and the peptide score after adjustment for baseline eGFR (*p* < 0.001), clinical score (*p* < 0.001) and individual clinically significant covariates (*p* < 0.001 for all), (*iii*) the significantly higher AUC of the peptide score (0.78 [0.73–0.83]) compared to that of the local clinical score (*p* < 0.001) or the baseline eGFR (*p* < 0.001), and (*iv*) the lack of improved performance when combining clinical and peptides-based scores (see below and Fig. S2). A peptide-based score above the threshold was associated with an increased risk of AKI (OR = 6.13 [3.95–9.59], *p* < 0.001; positive and negative predictive value 0.59 and 0.81, respectively, and positive net reclassification index (0.19) compared to clinical score Fig. 4), even after adjustment for the local clinical score or preoperative clinical features. Performances were also superior to the reference urinary biomarkers NGAL (0.70 [0.65–0.75], *p* = 0.004) and similar to the [IGFBP7]*[TIMP2] product (0.74 [0.68–0.79], *p* = 0.14). However, a [IGFBP7]*[TIMP2] product over the recommended cutoff of 0.3 was associated with an increased risk of AKI but with a lower prediction compared to the peptide-based score (OR 3.55 [2.28–5.65], *p* < 0.001). The urinary peptide score was also significantly associated with severe AKI stage 2 or 3, similarly to the [IGFBP7]*[TIMP2] product (AUC 0.74 [0.68–0.80] vs. 0.73 [0.67–0.79], respectively, *p* = 0.6), and outperformed NGAL (0.67 [0.61–0.74], *p* = 0.04).

The combination of the local clinical and the peptide-based scores did not significantly improve overall performances compared to the peptide-based score alone (Fig. 4). Last, the initial signature of 204 peptides could be reduced to 17 peptides using advanced feature selection methods such as genetic algorithms, with similar performances compared to the full 204 peptide signature in the validation cohort (AUC = 0.77 [0.72–0.82], *p* = 0.676 for comparison between the two AUC; OR = 5.67 [3.68–8.82], p < 0.001) (Additional file 1: Fig. S2 and Additional file 1: Table S2).

### Validation in an external cohort of patients admitted to the ICU

CBP surgery AKI is considered a standardized at-risk setting of conditions with the potential to induce AKI. To study the potential generalization of the use of peptide signatures in AKI, we also evaluated the performance of the urinary peptide signature in 1569 patients admitted to an intensive care unit (ICU), with various causes of AKI, including sepsis, unplanned surgery, and trauma (FROG-ICU cohort) [25–27].

**Fig. 5:**
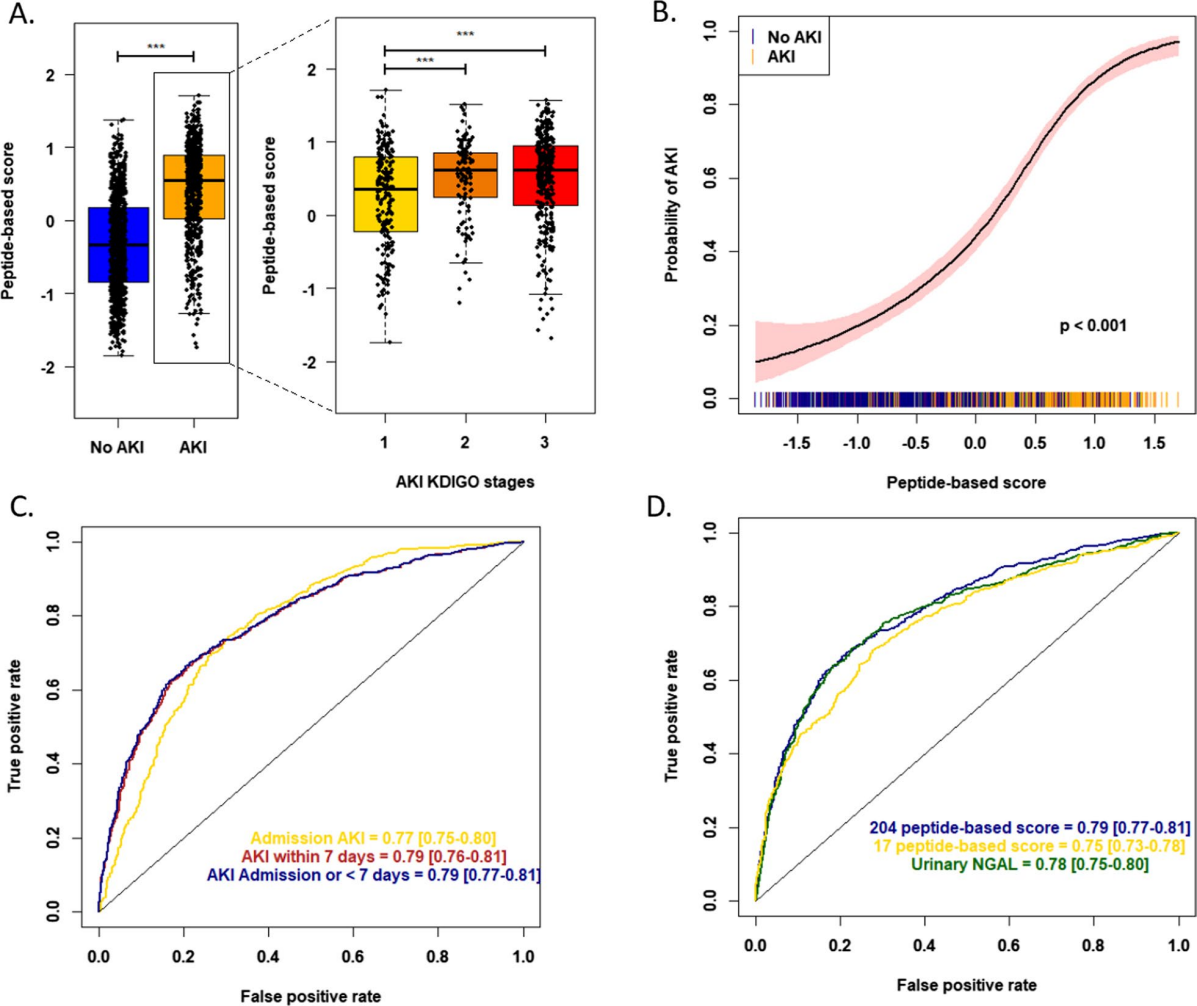
External validation of the predictive value of the urinary peptide signature for AKI diagnosis in an intensive care unit (ICU) cohort of 1,569 patients. **A** Peptide-based score according to AKI status (all stages (left part); according to KDIGO stages, right part) in the external ICU validation cohort. *** *p* < 0.001. **B** Spline plot of the association between the peptide-based score and the risk of developing AKI in the external ICU cohort. A univariate logistic generalized additive model was used. The black line indicates the estimated risk of AKI with respective 95% confidence intervals. The spikes show the distribution of the peptidome-based scores. **C** ROC curves and corresponding AUROC 95% confidence intervals of the 204 peptides-based score in the external ICU validation cohort, according to the time until AKI diagnosis. **D** ROC curves and corresponding AUROC 95% confidence intervals of the 204 peptides-based score and reference urinary biomarker NGAL in the external ICU validation cohort

Interestingly, the urinary peptide-based signature assessed in patients at their admission in the ICU was significantly associated with the development of AKI (Fig. 5), and its performance characteristics were equal to those obtained in the case of CBP surgery AKI (AUC 0.79 [0.77–0.81] versus 0.78 [0.73–0.83], *p* = 0.634; OR = 5.92 [4.73–7.45], *p* < 0.001). These findings were confirmed when patients were stratified according to the time until AKI diagnosis: diagnosis of AKI at admission AUC 0.77 [0.75–0.80]; AKI within 7 days of admission AUC 0.79 [0.76–0.81]. Subgroup analysis, according to the underlying disease that justified ICU admission, showed quite good performances among groups, despite some heterogeneity. The best AUCs were obtained in patients admitted after surgery (*n* = 146; AUC 0.86 [0.80–0.92]), for sepsis (*n* = 378; 0.78 [0.74–0.83]) or for impaired hemodynamic status (*n* = 193; AUC 0.81 [0.75–0.88]) (Additional file 1: Table S3).

When compared with reference urinary AKI biomarkers, such as NGAL, those performances were broadly equivalent (AUC = 0.78 [0.75–0.80], *p* = 0.426, Fig. 5) for both AKI at admission and AKI within seven days of admission (Additional file 1: Fig. S3). Evaluation of the [IGFBP7]*[TIMP2] product was not available in this cohort. It is worth noting that the reduced 17 peptide signature exhibited a significantly decreased performance compared to the full 204 peptide signature in this ICU setting of mixed AKI etiologies (AUC = 0.75 [0.73–0.78], *p* < 0.001).

### The urinary peptide-based AKI signature provides insights on early mortality

Among the 1170 patients of the CBP surgery cohort, 41 (3.5%) died during hospitalization, including 30 (2.5%) during the first month. The development of AKI was strongly associated with in-hospital mortality (OR 16.2 [7.5–40.1]; *p* < 0.001), even after adjustment of the preoperative mortality score (EuroSCORE-2: OR 13.8 [6.3–34.5]; *p* < 0.001) or after multivariate adjustment using a propensity score (OR 5.6 [2.4–14.8]; *p* < 0.001) (Additional file 1: Fig S4). In both the derivation and validation cohorts, the peptide-based score obtained in samples collected < 4 h after the surgery was associated with higher in-hospital mortality (AUC 0.77 [0.66–0.88]; *p* < 0.001; OR when peptidome score was above threshold: 6.2 [2.6–16.2]; *p* < 0.001). Similarly, in the external ICU validation cohort, the peptide-based score assessed at admission was also associated with the in-hospital mortality (OR 2.62 [2.05–3.38], *p* < 0.001).

## Discussion

In this study that included > 2400 patients, we identified a urinary peptide signature that predicts the development of AKI as early as 3–4 h after the initial insult in a variety of high-risk clinical situations, including CBP surgery and ICU admission. Cardiac surgery with cardiac bypass is the prototypical cause of AKI, with a pathophysiology based on various interrelated mechanisms (inflammation, ischemia, hemolysis, oxidative stress, tubular cell injury) [43]. The nature of the peptides identified can be clearly linked to these different mechanisms [8, 43–47] and potentially explains the good performance of the urinary peptide signature in predicting future AKI at this early stage. Furthermore, these mechanisms are shared by various other etiologies of AKI [8, 44–47], which may account for the excellent transferability of the urinary peptide signature to the ICU setting. Hence, our findings highly suggest that such an omic signature may help to refine, a posteriori, the molecular pathways that may predict the response to a dedicated therapeutic.

In the CBP surgery setting, the urine peptide signature outperformed all clinical parameters and urinary NGAL, while the predictive value of the peptide signature was similar to NGAL in an ICU setting. This is most likely due to differences in the time window of the initial event leading to the development of AKI. While in the CBP surgery cohort all samples were obtained at the latest 4 h after the initial injury, in the ICU validation cohort, urine samples were collected within 24 h after ICU admission. In addition, a number of ICU patients had already full-blown AKI on admission to the ICU [25, 27]. This more advanced stage of AKI in the ICU cohort may explain the comparable performance of the urine peptide signature and NGAL in this setting.

In the ICU cohort, the performance of the urine peptide signature was associated with a diagnosis warranting admission to ICU. As expected, because the peptides were identified in the CBP surgery setting, excellent performances were observed in the postoperative subgroup of ICU patients, but surprisingly also in the groups with hemodynamic failure or sepsis. In contrast, performance was poorer after cardiac arrest and respiratory failure, probably due to the involvement of other pathophysiological mechanisms.

A number of peptides in the signature can readily be linked to the pathophysiology of AKI (*e.g.*, alpha-1-antitrypsin (SERPINA1), calprotectin (S100A9), serum amyloid A (SAA) for inflammation, uromodulin (UMOD), albumin (ALB)). However, some other peptides, including thymosin β4 (TMSB4X), CD99, and Na–K-ATPase γ-subunit (FXYD2), are not currently known to be involved in AKI pathophysiology and may represent good candidates for further exploration of their role in AKI. Finally, the identified peptides also suggest candidate targets for specific treatment of AKI. For example, the increased urinary abundance of the complement factor B (CFB)-derived peptide in AKI argues for targeting the alternative complement pathway with recently developed complement inhibitors [48, 49]. We also observed here that calprotectin is dramatically induced in kidney after epithelial injury in a preclinical model. Targeting calprotectin signaling with paquinimod demonstrated beneficial effects to prevent the development of ischemic AKI [50]. This exemplifies the fact that such a urinary peptide-based strategy may, in addition to detecting at an early stage patients who will develop AKI, also furnish druggable targets.

The limitations of the direct clinical application of the peptide signature in this context of AKI are the specific equipment currently required (capillary electrophoresis coupled with mass spectrometry (CEMS)) and the time needed for the sample preparation and analysis (currently about 12 h). Future improvements on sample preparation, by avoiding the 6-h-long lyophilization step and reducing instrument time, should allow the analysis time window to be reduced to a few hours, compatible with the timely decision-making necessary in patients at risk for AKI. Alternatively, peptides could also be measured using strategies such as multiplex ELISA, if peptide abundance correlates with the parental protein abundance. Targeted mass spectrometry (*e.g.*, multiple reaction monitoring) is another alternative [51]. However, such strategies require a significant reduction in the number of peptides. We observed that reducing the signature from 204 to 17 peptides is possible without significant loss of performance in the CPB cohort, but it led to a significant reduction in the efficiency of AKI prediction in the ICU cohort. This may be due to the fact that the overall signature of 204 peptides incorporates information that is redundant in CBP-surgery-induced AKI, but is essential in other risk situations encountered in the ICU. As some peptides correlate well with clinical features (data not shown), one way to further reduce the size of the signature would be to identify a score complementary to the readily available clinical information. Finally, further improvement in the prediction and reduction in the number of urinary peptides could come from future studies focusing on the discovery of novel molecular markers in, for example, plasma, characterization of immune cell populations, and genetic predisposition for AKI in CBP surgery patients. Strong and complementary molecular markers identified from those sources could help, using machine learning, to further reduce the signature but also further improve the efficacy of the prediction.

Other limitations included the lack of assessment of the urinary [IGFBP7]*[TIMP2] product in all cohorts that would have allowed to robustly compare it to the peptide signature in clinical settings beyond CBP surgery. Also, we did not assess other single biomarkers like KIM-1 or L-FABP. However, the aim of our study was not only to test the AKI predictive value of urinary peptides but also to test whether this approach may identify new, targetable, molecular players of AKI to ultimately develop personalized medicine.

**In conclusion,** we have identified and validated a urine peptide signature predictive of AKI in a variety of situations at risk. This overarching signature, which contains numerous peptides directly associated with the pathophysiology of AKI, holds great promise for identifying patients developing AKI early after injury and developing tailored treatment.

## Supplementary Information

**Additional file 1: Supplementary Figure S1: S100A9 expression after epithelial injury.** **A**. Urinary calprotectin (S100A8/A9) abundance 4 hours after cardiac bypass-surgery. **B–F**. Blood urea nitrogen (B), mRNA *S100a9* (C), S100A9 immunostaining (D-E) and mRNA *Kim1* (F) in sham mice and after bilateral renal ischemia/reperfusion (hours 6, 24 and 48). **G–J**. mRNA expression of *S100a9* and *Kim1* in MCT cells submitted to interleukin-1b (IL1β, 10 ng/mL) or tumor necrosis factor-1a (TNFa, 10 ng/mL) (G-H) or hypoxia (I-J). *AKI*, acute kidney injury; *BUN*, blood urea nitrogen; *Norm*, normoxia; *Hyp*, hypoxia. **Supplementary Figure S2: Performances of the peptide-based signature to identify AKI that developed within the first 2 days following cardiac surgery.** ROC curves with corresponding AUROC and 95% confidence intervals of the local clinical score (blue, pointed), the full 204 peptides-based score (red), the urinary NGAL level (yellow, pointed) and the nephrocheck ([IGFBP7].[TIMP2] product) in the validation cohort. **Supplementary Figure S3: Reduction and combination of the peptide-based signature.** **A**. ROC curves with corresponding AUROC and 95% confidence intervals of the local clinical score (blue, pointed), the full 204 peptides-based score (red), the reduced 17 peptides-based score (black, dashed) and the combination of local clinical and full peptide-based score in the validation cohort. **B**. List of peptides included in the reduced 17-peptides signature according to their parental protein. *LMAN2*, Lectin mannose binding 2 ; *MGP*, Matrix gla protein. **Supplementary Figure S4: Performances of the 204 peptides-based signature and the reference urinary biomarker NGAL for AKI prediction in the external ICU validation cohort.** **A**. ROC curves with corresponding AUROC and 95% confidence intervals of the 204 peptides-based score and the reference urinary biomarker NGAL to predict AKI after ICU admission. **B**. ROC curves with corresponding AUROC and 95% confidence intervals of the 204 peptides-based score and reference urinary biomarker NGAL to predict the development of AKI within seven days after admission. **Supplementary Figure S5: Performances of the 204 peptides-based score for in-hospital mortality prediction.** Odds-ratio (OR) of in-hospital mortality were calculated with unadjusted, Euroscore-II-adjusted or propensity score-adjusted logistic regression. **Supplementary Table S1: Correlations between clinical characteristics and the 204-peptides-based score.** *BMI*, body mass index; *PAOD*, peripheral artery obliterans disease; *COPD*, chronic obstructive pulmonary disease; *LVEF*, left ventricular ejection fraction; *eGFR*, estimated glomerular filtration rate; *CBP*, cardiac bypass; *RBC*, red blood cells. **Supplementary Table S2: Performance of the peptide-based score to predict acute kidney injury in the external ICU validation cohort, according to the cause of admission to the intensive care unit.** *ICU*, intensive care unit; *AUROC*, area under the receiver operating characteristics curve. **Supplementary file S1: Methodology. Urinary peptidomics and statistical analyses.**

## Data Availability

All data, code, and materials used in the analysis are available upon request.

## Abbreviations

AKI: Acute kidney injury
CBP: Cardiopulmonary bypass
CKD: Chronic kidney disease
COPD: Chronic obstructive pulmonary disease
eGFR: Estimated glomerular filtration rate
KDIGO: Kidney diseases improving global outcomes
ICU: Intensive care unit
OR: Odds ratio
RBC: Red blood cells
