# Publisher Correction to: A universal predictive and mechanistic urinary peptide signature in acute kidney injury

**DOI:** 10.1186/s13054-022-04278-5

**Published:** 2022-12-29

**Authors:** Alexis Piedrafita, Justyna Siwy, Julie Klein, Amal Akkari, Ana Amaya-garrido, Alexandre Mebazaa, Anna Belen Sanz, Benjamin Breuil, Laura Montero Herrero, Bertrand Marcheix, François Depret, Lucie Fernandez, Elsa Tardif, Vincent Minville, Melinda Alves, Jochen Metzger, Kidney Attack Study Group, Etienne Grunenwald, Guylène Feuillet, Marie Buléon, Manon Brunet, Julia Grossac, Harald Mischak, Alberto Ortiz, Stéphane Gazut, Joost P. Schanstra, Stanislas Faguer

**Affiliations:** 1 Department of Nephrology and Organ Transplantation, University Hospital of Toulouse, and French Intensive Care Renal Network, 31000 Toulouse, France; 2 National Institute of Health and Medical Research (INSERM), UMR 1297, Institute of Cardiovascular and Metabolic Disease, 31000 Toulouse, France; 3 University Paul Sabatier, Toulouse-III, 31000 Toulouse, France; 4 Mosaiques Diagnostics GmbH, Hannover, Germany; 5 Université Paris-Saclay, CEA, List, 91120 Palaiseau, France; 6 Department of Anesthesiology, Critical Care and Burn Unit, Hôpitaux Universitaires Saint Louis-Lariboisière, Assistance Publique-Hôpitaux de Paris, Université Paris Diderot-Paris 7, Sorbonne Paris Cité, UMR-S 942, INSERM, France, INI-CRCT, ParisNancy, France; 7 School of Medicine, IIS-Fundación Jiménez Díaz, Autonomous University of Madrid, FRIAT and REDINREN, Madrid, Spain; 8 Department of Cardiac and Vascular Surgery, University Hospital of Toulouse, 31000 Toulouse, France; 9 Department of Anesthesiology and Critical Care Medicine, University Hospital of Toulouse, 31000 Toulouse, France

**Correction to: Crit Care 26, 344 (2022)**. 10.1186/s13054-022-04193-9

Following publication of the original article [1], the authors identified errors in the authorship which was caused by the publisher. Three of the collaborating authors were incorrectly listed as authors.

The authorship has been updated in this Publisher Correction article and the original article [1] has been corrected. The publisher apologises to the authors and readers for the inconvenience caused by these mistakes.
