# Supplementary material for: A universal predictive and mechanistic urinary peptide signature in acute kidney injury
Source: Crit Care. 2022 Nov 7;26:344. doi: 10.1186/s13054-022-04193-9 (PMC9640896; doi:10.1186/s13054-022-04193-9)
Supplement: Supplementary file 1 — Additional file 1: Supplementary Figure S1: S100A9 expression after epithelial injury. A. Urinary calprotectin (S100A8/A9) abundance 4 hours after cardiac bypass-surgery. B–F. Blood urea nitrogen (B), mRNA S100a9 (C), S100A9 immunostaining (D-E) and mRNA Kim1 (F) in sham mice and after bilateral renal ischemia/reperfusion (hours 6, 24 and 48). G–J. mRNA expression of S100a9 and Kim1 in MCT cells submitted to interleukin-1b (IL1β, 10 ng/mL) or tumor necrosis factor-1a (TNFa, 10 ng/mL) (G-H) or hypoxia (I-J). AKI, acute kidney injury; BUN, blood urea nitrogen; Norm, normoxia; Hyp, hypoxia. Supplementary Figure S2: Performances of the peptide-based signature to identify AKI that developed within the first 2 days following cardiac surgery. ROC curves with corresponding AUROC and 95% confidence intervals of the local clinical score (blue, pointed), the full 204 peptides-based score (red), the urinary NGAL level (yellow, pointed) and the nephrocheck ([IGFBP7].[TIMP2] product) in the validation cohort. Supplementary Figure S3: Reduction and combination of the peptide-based signature. A. ROC curves with corresponding AUROC and 95% confidence intervals of the local clinical score (blue, pointed), the full 204 peptides-based score (red), the reduced 17 peptides-based score (black, dashed) and the combination of local clinical and full peptide-based score in the validation cohort. B. List of peptides included in the reduced 17-peptides signature according to their parental protein. LMAN2, Lectin mannose binding 2 ; MGP, Matrix gla protein. Supplementary Figure S4: Performances of the 204 peptides-based signature and the reference urinary biomarker NGAL for AKI prediction in the external ICU validation cohort. A. ROC curves with corresponding AUROC and 95% confidence intervals of the 204 peptides-based score and the reference urinary biomarker NGAL to predict AKI after ICU admission. B. ROC curves with corresponding AUROC and 95% confidence intervals of the 204 peptides-based s [file 13054_2022_4193_MOESM1_ESM.docx]

**Supplementary Figure S1**: **S100A9 expression after epithelial injury**. **A.** Urinary calprotectin (S100A8/A9) abundance 4 hours after cardiac bypass-surgery. **B-F** Blood urea nitrogen (B), mRNA *S100a9* (C), S100A9 immunostaining (D-E) and mRNA *Kim1* (F) in sham mice and after bilateral renal ischemia/reperfusion (hours 6, 24 and 48). **G-J.** mRNA expression of *S100a9* and *Kim1* in MCT cells submitted to interleukin-1β (IL1β, 10 ng/mL) or tumor necrosis factor-1α (TNFα, 10 ng/mL) (G-H) or hypoxia (I-J). *AKI*, acute kidney injury; *BUN*, blood urea nitrogen; *Norm*, normoxia; *Hyp*, hypoxia.

**Supplementary Figure S2: Reduction and combination of the peptide-based signature. A**. ROC curves with corresponding AUROC and 95% confidence intervals of the local clinical score (blue, pointed), the full 204 peptides-based score (red), the reduced 17 peptides-based score (black, dashed) and the combination of local clinical and full peptide-based score in the validation cohort. **B.** List of peptides included in the reduced 17-peptides signature according to their parental protein. *LMAN2 :* Lectin mannose binding 2 *; MGP :* Matrix gla protein*.*


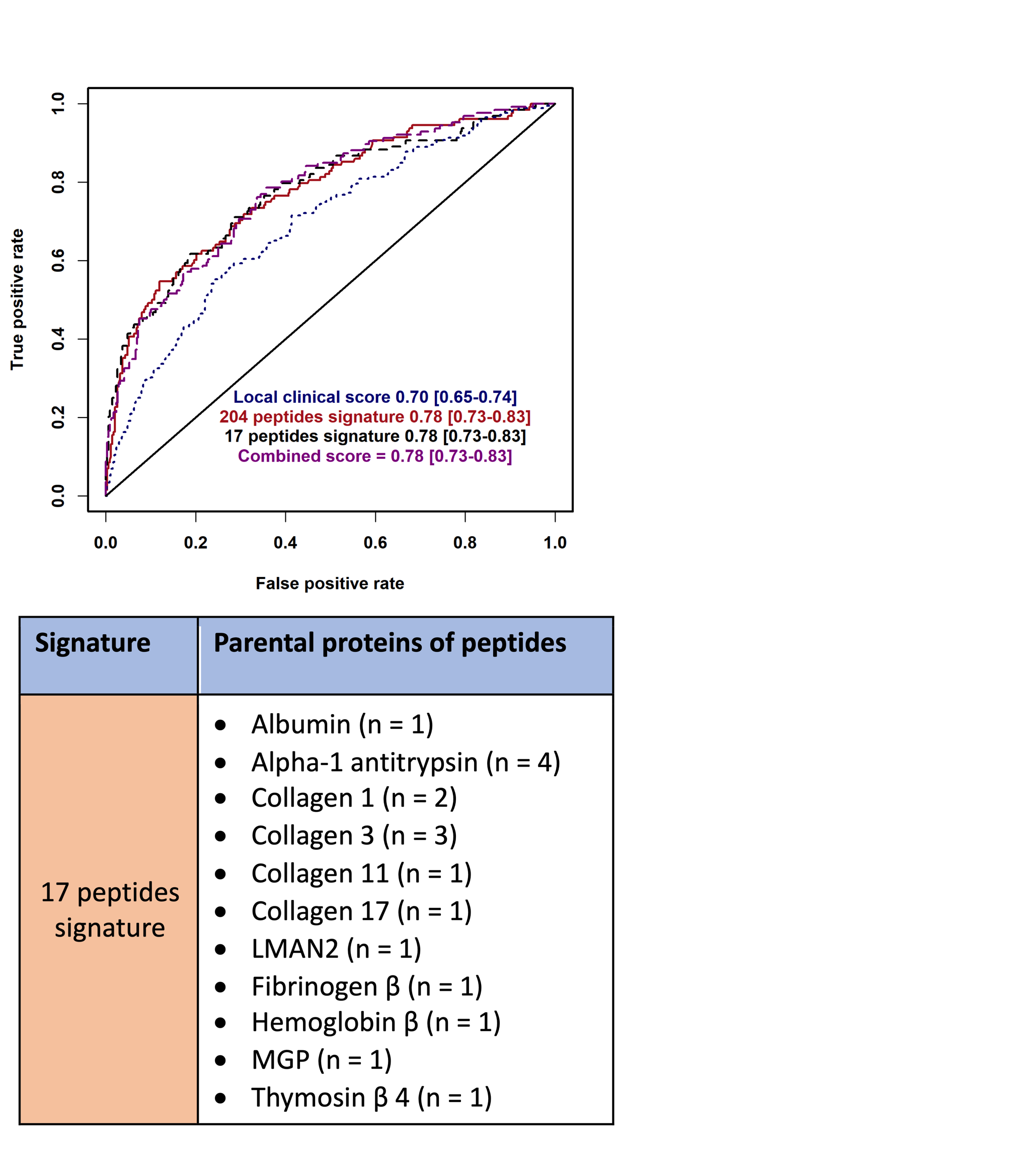


**Supplementary Figure S3: Performances of the 204 peptides-based signature and the reference urinary biomarker NGAL for AKI prediction in the external ICU validation cohort. A.** ROC curves with corresponding AUROC and 95% confidence intervals of the 204 peptides-based score and the reference urinary biomarker NGAL to predict AKI after ICU admission. **B.** ROC curves with corresponding AUROC and 95% confidence intervals of the 204 peptides-based score and reference urinary biomarker NGAL to predict the development of AKI within seven days after admission.

**
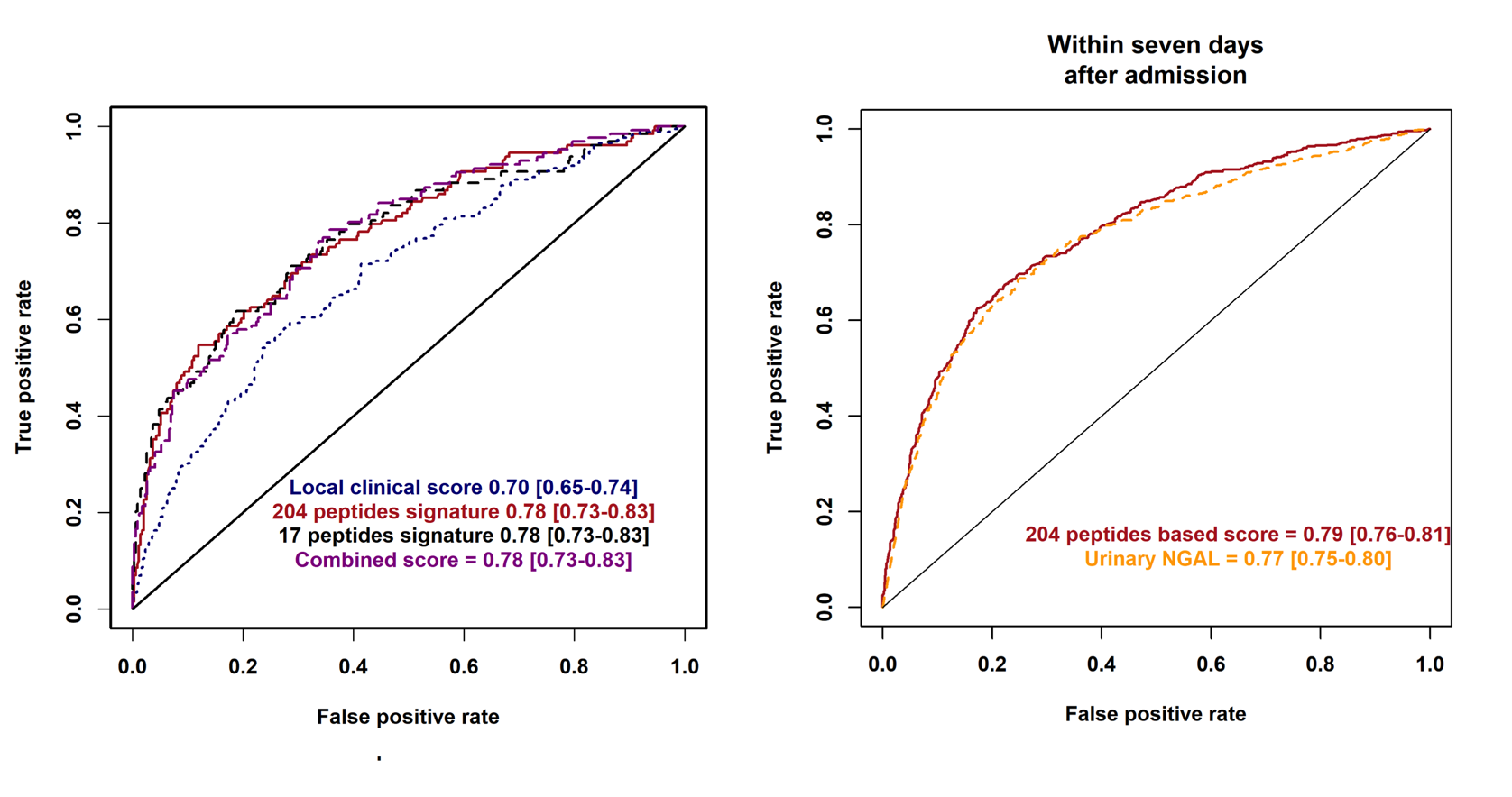
**

**Supplementary Figure S4: Performances of the 204 peptides-based score for in-hospital mortality prediction.** Odds-ratio (OR) of in-hospital mortality were calculated with unadjusted, Euroscore-II-adjusted or propensity score-adjusted logistic regression.

**
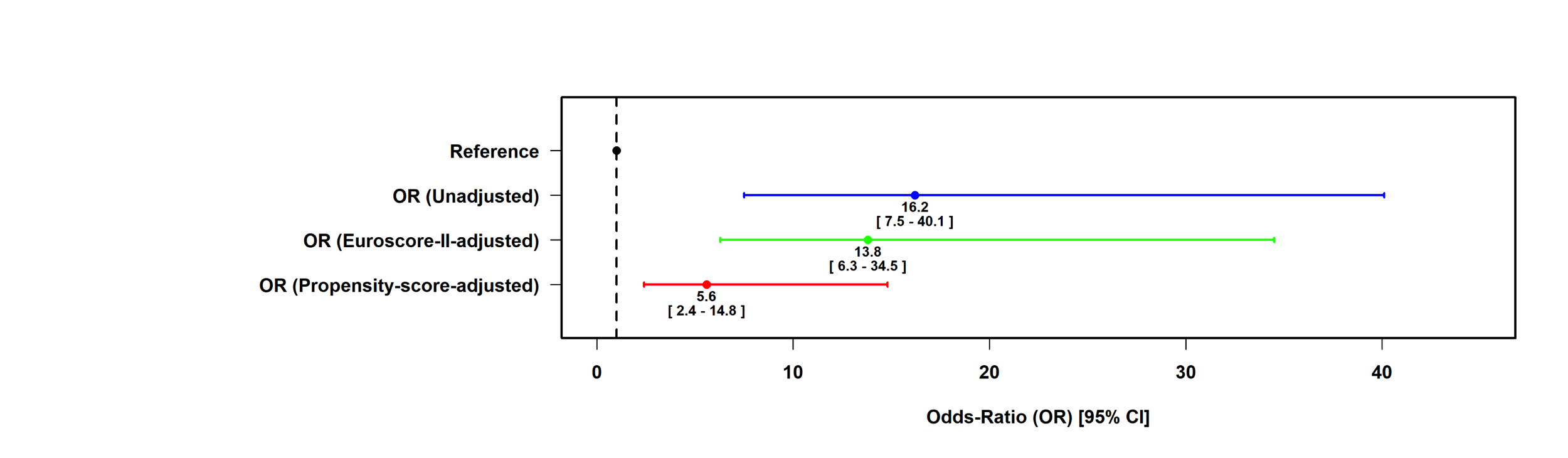
**

**Supplementary Table S1**: **Correlations between clinical characteristics and the 204-peptides-based score.** *BMI*, body mass index; *PAOD*, peripheral artery obliterans disease; *COPD*, chronic obstructive pulmonary disease; *LVEF*, left ventricular ejection fraction; *eGFR*, estimated glomerular filtration rate; *CBP*, cardiac bypass; *RBC*, red blood cells.

| **Variables** | **Univariate adjusted-p-values** | **Multivariate p-values** |
| --- | --- | --- |
| **Sex** | 0.202 | - |
| **Age** | < 0.001 | 0.515 |
| **BMI** | 0.074 | - |
| **Diabetes** | 0.014 | 0.033 |
| **Endocarditis** | 0.058 | - |
| **Hypertension** | < 0.001 | 0.168 |
| **PAOD** | 0.104 | - |
| **Stroke** | 0.125 | - |
| **COPD** | 0.033 | 0.034 |
| **LVEF** | 0.496 | - |
| **eGFR** | < 0.001 | < 0.001 |
| **Kidney graft recipient** | 0.095 | - |
| **Surgery type** | < 0.001 | < 0.001 |
| **Previous cardiac surgery** | 0.035 | 0.016 |
| **CBP time** | 0.006 | 0.007 |
| **RBC yes/no** | 0.006 | 0.062 |
| **Packed RBC number** | 0.006 | 0.370 |

**Supplementary Table S2: weighted score of each peptide included in the 17-peptides signature**. AUROC, area under the receiver operating characteristics curve.

| Rank | AUROC | Protein origin |
| --- | --- | --- |
| 1 | 0.73 | ALB |
| 2 | 0.62 | SERPINA1 |
| 3 | 0.68 | COL3A1 |
| 4 | 0.77 | SERPINA1 |
| 5 | 0.60 | LMAN2 |
| 6 | 0.59 | COL11A2 |
| 7 | 0.63 | FGB |
| 8 | 0.63 | SERPINA1 |
| 9 | 0.60 | HBA1 |
| 10 | 0.74 | COL1A1 |
| 11 | 0.61 | COL17A1 |
| 12 | 0.64 | COL3A1 |
| 13 | 0.62 | MGP |
| 14 | 0.71 | SERPINA1 |
| 15 | 0.65 | COL1A2 |
| 16 | 0.64 | COL3A1 |
| 17 | 0.59 | TMSB4X |

**Supplementary Table S3: Performance of the peptide-based score to predict acute kidney injury in the external ICU validation cohort, according to the cause of admission to the intensive care unit**. ICU, intensive care unit; AUROC, area under the receiver operating characteristics curve.

**Supplementary File S1**: **Methodology. Urinary peptidomics and statistical analyses.**

***Clinical characteristics: description and comparisons***

For clinical descriptive analysis, missing data were removed (pairwise deletion) and data were not transformed prior to analysis. Quantitative variables were presented as mean ± SD and qualitative variables as number and percentage. Two-group comparisons were conducted using the Aspin-Welch, Chi2 or Fisher Exact tests, as appropriate. Multivariate analysis was based on logistic regression and includes all pre-operative variables with univariate adjusted p-values < 0.1 (events per variable > 10). Models qualities of the logistic regressions were verified with the Hosmer-Lemeshow test. Survival was estimated using Kaplan-Meier curves with a log-rank test for two-group comparison. For in-hospital mortality analysis, odds ratios (OR) were extracted from univariate (unadjusted) or multivariate (adjusted) logistic regression models using confounders as covariates. In the CBP-surgery derivation cohort, adjustment was done using as covariate the mortality score Euroscore-II or a logistic propensity score based on clinical variables identified as being associated with in-hospital mortality using univariate adjusted testing. In the external ICU validation cohort, adjustments were made for age, sex and diagnosis.

***Clinical scores***

For the development of the clinical score, all patients from the CBP-surgery discovery cohort were considered (n = 509). A set of 14 variables (age, gender, body mass index, diabetes mellitus, endocarditis, hypertension, peripheric artery obliterative disease, stroke, chronic obstructive pulmonary disease, left ventricle ejection fraction, eGFR, kidney transplant recipient, second or third cardiac surgery, kind of surgery, CPB duration, number of packed red blood cells) was first used, and a simple logistic regression on a reduced variable set obtained by stepwise selection was built (caret, “glmStepAIC,” repeated cross-validation number = 10/repeats = 3, “ROC” metric). The final model used only six easily available features (age, hypertension, eGFR (CKD-Epi), kidney transplant recipient, valvular surgery and CPB duration). For clinical score validation, all patients from the CBP-surgery validation cohort except eight with missing data (n = 653) were considered. Results were extracted as probability scores from both cohorts and scores were calculated as logit of those probabilities. Goodness of fit was verified using a Hosmer-Lemeshow test and an observed-predicted plot. Events per variable were verified to be above 10.

***Peptidome analysis of urines***

Briefly, urines were defrosted and 1:1 diluted with 700 μL of a solution containing 2 M urea, 0.1 M NaCl, 10 mM NH4OH and 0.02 % SDS. The mixture was then filtered through a 20 kDa MW cut-off ultracentrifugation filter device (Sartorius, Göttingen, Germany) at 3000 × g for 1 hour at 4 °C to eliminate high molecular weight proteins. Obtained filtrate was loaded onto a pre-equilibrated PD-10 desalting column (GE Healthcare, Uppsala, Sweden) and eluted using 0.01 % aqueous NH_4_OH. The eluate was subsequently freeze-dried and stored at 4 °C prior to being resuspended in 10 mL HPLC-grade water for CE-MS analysis.

CE-MS analysis was performed as described using a P/ACE MDQ capillary electrophoresis system (Beckman Coulter, Fullerton, CA, USA) on line coupled to a MicroTOF MS (Bruker Daltonic, Bremen,Germany). Samples were injected hydrodynamically at 2.0 psi for 99 sec (ca. 250 nL) and separation of peptides was achieved by reverse polarity at 25 kV for the first 30 min, and with increasing pressure (up to 0.5 psi) for another 34 min. The cartridge temperature was maintained at 25 °C. Running. A solution of 20% acetonitrile (Sigma-Aldrich, Taufkirchen, Germany) in HPLC-grade water (Roth, Karlsruhe, Germany) supplemented with 0.94% formic acid (Sigma-Aldrich) was used as running buffer. The ESI sprayer (Agilent Technologies, Palo Alto, CA, USA) was grounded, and the ion spray interface potential was set between -4 and -4.5 kV. Spectra were accumulated every 3 seconds.

After the CE-MS analysis, mass spectral ion peaks representing identical molecules at different charge states were deconvoluted into single masses using MosaiquesVisu software (52). Reference signals of 1770 urinary polypeptides were used for CE time calibration by local regression. For normalization of analytical and urine dilution variances, MS signal intensities were normalized relative to 29 internal standard peptides using linear regression (53). The obtained peak lists characterized each polypeptide by its molecular mass (in Daltons), normalized CE migration time (in minutes) and normalized signal intensity. Sample-specific peptide lists were deposited in a SQL database for subsequent statistical analysis.

Candidate biomarkers were sequenced using CE-MS/MS or LC-MS/MS analysis, as described in detail before (54). In brief, Ultimate 3000 nano-flow system (Dionex/LC Packings, USA) or a P/ACE MDQ capillary electrophoresis system (Beckman Coulter, Fullerton, CA), both connected to an LTQ Orbitrap hybrid mass spectrometer (Thermo Fisher Scientific, Germany) equipped with a nano-electrospray ion source were used for MS/MS experiments. The mass spectrometer was operated in data-dependent mode to automatically switch between MS and MS/MS acquisition. Survey full-scan MS spectra (from m/z 300–2,000) were acquired in the Orbitrap. Ions were sequentially isolated for fragmentation. Data files were searched against the UniProt human nonredundant database using Proteome Discoverer 2.4 and the SEQUEST search engine. Relevant settings were: no fixed modifications, oxidation of methionine and proline as variable modifications. The high-confidence peptides were defined by cross-correlation (Xcorr) >1.9 and rank = 1. Precursor mass tolerance was 5 ppm and fragment mass tolerance was 0.05 Da. For further validation of obtained peptide derivations, the correlation between peptide charge at the working pH of 2 and CE-migration time was utilized to minimize false-positive derivation rates: calculated CE-migration time of the sequence candidate based on its peptide sequence (number of basic amino acids) was compared to the experimental migration time (55).

***Peptide-based score***

For peptidome analysis, among the 5862 peptides, only peptides with less than 70% of missing data in at least one group were considered for analysis, resulting in a set of 1255 peptides. For those peptides, missing values were replaced by 0 before further analysis. Univariate testing between AKI and non-AKI patients was performed using the Wilcoxon signed-rank test, followed by Benjamini-Hochberg false discovery rate adjustment. Correlations were performed according to the Pearson method.

For score derivation, all patients from the CBP-surgery discovery cohort with available peptidome data were considered (n = 446). A set of 328 differentially abundant peptides with a significant Benjamini-Hochberg-adjusted Wilcoxon signed-rank testing was identified in this cohort. The amino acid sequence could be obtained for 204 peptides of the 328 peptides. These 204 peptides set was used to build the main Support Vector Machine-based predictor (MosaCluster software (37)). Results were extracted as a calibrated score based on derivation. Score performances in the CBP-surgery derivation cohort were estimated using the leave-one-out procedure. Score validation was then obtained in the CBP-surgery validation cohort in patients with available peptidome data; n = 480. External validation in the ICU context was obtained on all patients from the FROG-ICU cohort with available AKI status and peptidome data (n = 1569).

***Comparisons between scores***

Performances of the various scores were evaluated with:

- Quantitative univariate testing according to AKI status (Aspin-Welch Test).
- Logistic univariate and multivariate generalized additive models for AKI prediction adjusted for clinical score, eGFR or pre/per-operative clinical variables significantly associated with AKI using multiple smooth regression (mgcv package).
- Area under receiver operating characteristic (AUROC) obtained using the ROCR and pROC packages. Confidence intervals (CI) were calculated using the DeLong method. Results were presented as AUC [95% confidence intervals]. Comparison between ROC curves were performed using either paired Delong tests or unpaired Delong tests, as needed.
- Odds ratios from univariate or multivariate logistic regression, using scores as a binary variable according to chosen threshold. Tests were conducted in all available individuals in the corresponding dataset. The regression was adjusted for clinical variables only for the peptidomic score (events per variable > 10). Optimal thresholds for each score were chosen using the best Youden index (Sensitivity - (1-Specificity)) in the derivation cohort: local clinical score threshold : -1.159 ; full 204 peptides based threshold : -0.027; Combined score threshold : 0.157 ; Urinary NGAL threshold : 19.165 μg/g.

For all analyses, adjusted p-values < 0.05 were considered significant.

***Correlations between peptides signature and clinical characteristics***

Univariate association with continuous clinical parameters were performed using the Spearman correlation test. Univariate association with clinical qualitative features was performed using Wilcoxon or Kruskal-Wallis tests, as appropriate. A multivariate model was constructed using generalized additive model smooth multiple regression (mgcv R package).

***Combination of clinical and peptides-based scores***

For combination of clinical and peptidomic scores, all patients with available clinical and peptidome data from validation cohort (n = 474) were considered. The clinical score was used as AKI probability extracted from the glm model. The peptide-based score was calibrated as a probability using logistic smooth regression built in the derivation cohort. Combination was achieved by geometric mean calculation of clinical and peptidomic calibrated probabilities. Other combination methods (addition, logistic regression based on scores/probabilities) were tried without further improvement in prediction performances, and thus were not shown in the manuscript.

***Genetic algorithm***

Genetic algorithm is a stochastic method that can be used in the context of combinatorial optimization. The objective was to find one of the best subsets of peptides by considering larger amounts of initial ones without testing all the combinations. A fitness function evaluated the relevance of each signature in the optimization procedure. By these algorithms inspired by the natural evolution process, signatures evolve over successive iterations towards better and better solutions adapted to the AKI prediction task. The genetic algorithm analyses were performed on the derivation cohort with the R package GA (42). To evaluate the signatures during the optimization process we used, as the fitness value, the AUC ROC measure of the AKI prediction obtained by a linear SVM model built on the signature under a 3-fold cross validation. This was applied to the entire peptides dataset to identify reduced signature of peptides predictive of the AKI risk.

**Ranking of peptides included in the reduced signature**

To approximate the importance of each predictor, a filtering method was used. For classification, ROC curve analysis was conducted on each predictor. For 2-class problems, a series of cutoff was applied to the predictor data to predict the class. The sensitivity and specificity were computed for each cutoff and the ROC curve was computed. The trapezoidal rule was used to compute the AUROC. This aera was used as the measure of variable importance. For multi-class outcomes, the problem was decomposed into all pair-wise problems and the aera under the curve was calculated for each class pair. For a specific class, the maximum AUC across the relevant pair-wise AUC’s was used as the variable importance measure.

**Mouse model of ischemic AKI**

C57Bl/6 male mice (n=5-9; 8 weeks old; Janvier lab, France) were sedated using isoflurane. Median laparotomy was performed followed by bilateral clamping of renal arteries for 20 minutes. Mice were euthanized following an intraperitoneal pentobarbital injection (180 μg/g) at 6, 24 and 48 hours. Blood urea nitrogen (BUN) was measured at each time point. Samples of the left kidney were collected and thawed in liquid nitrogen or embedded in paraffin. Expression of *Kim1* and *S100A9* genes was assessed by quantitative polymerase chain reaction (QuantStudio Design & Analysis Software v.1.5.1 with ONEGreen FAST qPCR Premix, OZYME) of the mRNA extracted using the RNA Easy Minikit (Qiagen). The housekeeping gene *Hprt* was used as a reference gene and the relative mRNA fold changes between groups were calculated using the 2−ΔΔCt method.

Formalin fixed kidneys were embedded in paraffin, sectioned in 4 μm thick slices (whole kidney) and used for immunohistochemistry. Goat anti-mouse S100A9 (1/100^e^, Bio-techne) was incubated for 1h at room temperature. Following this, the specimens were washed twice with TBS 0,1% Tween 20 and incubated with Histofine simple stain MAX-PO (goat, Nichirei, Tokyo, Japan) for 1h and revelation was made with substrate Dako Envision system (K4010; Dako, les Ulis, France). Finally, sections were counterstained with hematoxylin then dehydrated and mounted. Negative controls for the immunohistochemical procedures included substitution of the primary antibody with non-immune sera. Sections were scanned using a Nanozoomer 2.0 RS (Hamamatsu Photonics SARL, Massy, France).

**Cell culture**

MCT cells were grown under standard conditions (21% O_2_, 5% CO_2_, 37°C) with DMEM high glucose Glutamax medium (Gibco 61965-026), supplemented with penicillin-streptomycin (1%, Sigma P0781) and 10% calf serum (Gibco FBS One Shot A31608-01). For hypoxia experiments, MCT cells were exposed for 48h to either hypoxia (1% O2, 5% CO2, 37°C) or normoxia (21% O2, 5% CO2, 37°C). For inflammatory cytokine experiments, after 24h FBS starvation, MCT cells were treated with either 10ng/mL recombinant murine TNFα (Gibco) or 10ng/mL recombinant murine IL1β (Preprotech) for 8h. Expression of *Kim1* and *S100A9* genes was assessed by quantitative polymerase chain reaction (QuantStudio Design & Analysis Software v.1.5.1 with ONEGreen FAST qPCR Premix, OZYME) of the mRNA extracted using the RNA Easy Minikit (Qiagen). The housekeeping gene *Hprt* was used as a reference gene and the relative mRNA fold changes between groups were calculated using the 2−ΔΔCt method.
